# The Association between the Alcohol Biomarker Phosphatidylethanol (PEth) and Self-Reported Alcohol Consumption among Russian and Norwegian Medical Patients

**DOI:** 10.1093/alcalc/agab013

**Published:** 2021-03-03

**Authors:** Benedicte Jørgenrud, Saranda Kabashi, Aleksei Nadezhdin, Evgeny Bryun, Evgenya Koshkina, Elena Tetenova, Anners Lerdal, Gudmund Norby, Alexey Kolgashkin, Alexei Petukhov, Sergey Perekhodov, Elena Davydova, Vigdis Vindenes, Danil Gamboa, Stig Tore Bogstrand

**Affiliations:** Department of Forensic Sciences, Oslo University Hospital, P.O. Box 4950 Nydalen, N-0424 Oslo, Norway; Department of Forensic Sciences, Oslo University Hospital, P.O. Box 4950 Nydalen, N-0424 Oslo, Norway; Institute of Health and Society, Faculty of Medicine, University of Oslo, P.O. Box 1130 Blindern, N-0318 Oslo, Norway; Moscow Research and Practical Centre on Addictions of Moscow, Department of Public Health, 109390, Ljublinskaya ul. 37/1, Moscow, Russia; Russian Medical Academy of Continuous Professional Education, 125993, Barrikadnaya ul. 2/1, str. 1, Moscow, Russia; Moscow Research and Practical Centre on Addictions of Moscow, Department of Public Health, 109390, Ljublinskaya ul. 37/1, Moscow, Russia; Russian Medical Academy of Continuous Professional Education, 125993, Barrikadnaya ul. 2/1, str. 1, Moscow, Russia; Moscow Research and Practical Centre on Addictions of Moscow, Department of Public Health, 109390, Ljublinskaya ul. 37/1, Moscow, Russia; Moscow Research and Practical Centre on Addictions of Moscow, Department of Public Health, 109390, Ljublinskaya ul. 37/1, Moscow, Russia; Institute of Health and Society, Faculty of Medicine, University of Oslo, P.O. Box 1130 Blindern, N-0318 Oslo, Norway; Research Department, Lovisenberg Diaconal Hospital, P.O. Box 04970, Nydalen N-0440 Oslo, Norway; Medical Department, Lovisenberg Diaconal Hospital, P.O. Box 04970, Nydalen N-0440 Oslo, Norway; Moscow Research and Practical Centre on Addictions of Moscow, Department of Public Health, 109390, Ljublinskaya ul. 37/1, Moscow, Russia; Moscow Research and Practical Centre on Addictions of Moscow, Department of Public Health, 109390, Ljublinskaya ul. 37/1, Moscow, Russia; Sechenov First Moscow State Medical University, 119991, Bolshaya Pirogovskaya ul. 2, str. 4, Moscow, Russia; Demikhov Moscow Clinical Hospital, 109263, Shkuljova ul. 4, str. 1, Moscow, Russia; Demikhov Moscow Clinical Hospital, 109263, Shkuljova ul. 4, str. 1, Moscow, Russia; Department of Forensic Sciences, Oslo University Hospital, P.O. Box 4950 Nydalen, N-0424 Oslo, Norway; Norwegian Centre for Addiction Research, University of Oslo, P.O. Box 1130 Blindern, N-0318 Oslo, Norway; Department of Forensic Sciences, Oslo University Hospital, P.O. Box 4950 Nydalen, N-0424 Oslo, Norway; Institute of Health and Society, Faculty of Medicine, University of Oslo, P.O. Box 1130 Blindern, N-0318 Oslo, Norway; Medical Department, Lovisenberg Diaconal Hospital, P.O. Box 04970, Nydalen N-0440 Oslo, Norway; Department of Forensic Sciences, Oslo University Hospital, P.O. Box 4950 Nydalen, N-0424 Oslo, Norway; Institute of Health and Society, Faculty of Medicine, University of Oslo, P.O. Box 1130 Blindern, N-0318 Oslo, Norway

## Abstract

**Aims:**

Valid measures to identify harmful alcohol use are important. Alcohol Use Disorders Identification Test (AUDIT) is a validated questionnaire used to self-report harmful drinking in several cultures and settings. Phosphatidylethanol 16:0/18:1 (PEth) is a direct alcohol biomarker measuring alcohol consumption levels. The aim of this study was to investigate how PEth levels correlate with AUDIT-QF and weekly grams of alcohol consumed among patients in two urban hospitals. In addition, we wanted to investigate the predictive value of PEth in identifying harmful alcohol use as defined by AUDIT-QF and weekly grams of alcohol cutoffs.

**Methods:**

A cross-sectional study comprising acute medically ill patients with measurable PEth levels (≥0.030 μM) admitted to two urban hospitals in Oslo, Norway (N = 931) and Moscow, Russia (N = 953) was conducted using PEth concentrations in whole blood, sociodemographic data and AUDIT-QF questionnaires.

**Results:**

PEth levels from patients with measurable PEth were found to be positively correlated with AUDIT-QF scores, with PEth cutpoints of 0.128 μM (Oslo) and 0.270 μM (Moscow) providing optimal discrimination for harmful alcohol use defined by AUDIT-QF (the difference between cities probably reflecting different national drinking patterns in QF). When converting AUDIT-QF into weekly grams of alcohol consumed, the predictive value of PEth improved, with optimal PEth cutpoints of 0.327 (Oslo) and 0.396 (Moscow) μM discriminating between harmful and non-harmful alcohol use as defined in grams (≥350 grams/week).

**Conclusions:**

By using PEth levels and converting AUDIT-QF into weekly grams of alcohol it was possible to get an improved rapid and sensitive determination of harmful alcohol use among hospitalized patients.

## Teaser

**Short Summary**: AUDIT-QF among acute medically ill patients was found to be positively correlated with the alcohol biomarker PEth, with improved predictive value of PEth when converting AUDIT-QF to weekly grams of alcohol. PEth may be used to discriminate harmful alcohol use in clinical settings.

## INTRODUCTION

Alcohol use might be a risk factor for more than 30 diseases (defined by ICD-10) (World Health Organization, 2007), and is also associated with intentional and unintentional injuries (Room et al., 2005) and premature death (Griswold et al., 2018). In a study by Wood et al. (2018), they found that drinking ≥350 grams alcohol/week at the age of 40 years decrease your life expectancy by 4–5 years compared to those drinking 0–100 grams alcohol/week (Wood et al., 2018). In addition, patients with high alcohol consumption generally have a higher risk of reduced compliance to medical treatment (Bryson et al., 2008). As even quite moderate alcohol intake may impact the health of a patient, a correct assessment of alcohol intake is of clinical importance, although this is rarely done in hospital settings.

Identification of harmful alcohol use can be done using self-report questionnaires. These are easy and noninvasive, and they can be performed in most environments and settings and without dedicated personnel. The Alcohol Use Disorders Identification Test (AUDIT) is a validated screening test for alcohol use and harmful drinking the last 12 months (Babor et al., 2001). In clinical settings such as emergency departments, a brief measure, such as AUDIT-C, a short version of AUDIT consisting of the first three questions, could be used to assess the patient’s alcohol consumption. Each question has five response alternatives, which are given points from 0 to 4, depending on how frequent or how many units are consumed. Higher scores indicate higher consumption but may also reflect different drinking pattern. One drawback for AUDIT, as for all self-report questionnaires, is the risk of underreporting or overreporting because of social desirability (Boniface et al., 2014; Stockwell et al., 2014, 2016) or recall bias. In addition, AUDIT in its original form does not procure exact quantification on alcohol consumed, merely an identification of harmful use and drinking pattern.

In addition to questionnaires, the potential use of alcohol biomarkers is widespread, i.e. to assess alcohol use in DUI-suspected drivers, in hospitals to assess the amount of alcohol intake and the risk of developing delirium tremens, and in population health surveys. Several traditional biomarkers can identify harmful alcohol use, and these include direct and indirect markers (Conigrave et al., 2002; Musshoff, 2002; Golka and Wiese, 2004; Bortolotti et al., 2006; Hoiseth et al., 2009; Favretto et al., 2010; Litten et al., 2010; Andresen-Streichert et al., 2018). Phosphateidylethanol (PEth) 16:0/18:1 is a promising biomarker which can quantify long-term alcohol. PEth is a lipid species in the cell wall formed in the presence of ethanol, catalyzed by the enzyme phospholipase D (Kobayashi and Kanfer, 1987; Gustavsson, 1995). From previous studies, the formation rate of PEth in human blood appears to be correlated with alcohol consumed, and PEth concentration is affected by quantity, frequency and recency of alcohol consumed (Ulwelling and Smith 2018). PEth elimination rate has been determined in patients with alcohol dependence in detoxificiation programs to be 3–5 days after sobriety (Varga et al., 2000; Wurst et al., 2010), although elimination rates appear to vary (Gnann et al., 2012). The advantage of using PEth over other biomarkers for ethanol is that it does not appear to be affected by factors such as age, gender and non-alcohol-related disease (Stewart et al., 2009; Wurst et al., 2010).

Several studies have investigated the relationship between AUDIT and PEth, and found significant correlations (Francis et al., 2015; Piano et al., 2015; Schrock et al., 2017). However, both quantity and frequency of alcohol will affect PEth concentration, and it is possible to calculate the weekly grams of alcohol consumed from the first two questions of the AUDIT. Harmful alcohol use is a commonly known risk factor for several diseases, but alcohol screening is rarely done in clinical settings, and few studies have investigated the association between self-reported alcohol intake and the biomarker PEth in hospital populations. Because of the sociocultural differences between Norway and Russia, we wanted to investigate the performance of the measures separately in two different patient populations, using the same study design, and compare the measures between the two sites. As PEth is a direct biomarker, its formation is related to level of alcohol consumption, and not the other dimensions of the full AUDIT. Therefore, we limited the analysis to the first two items of AUDIT (AUDIT-QF), drinking pattern and quantity consumed, corresponding to the performance of PEth in identifying harmful alcohol use.

The aim of this study was to investigate if levels of the alcohol biomarker PEth in blood correlate with self-reported alcohol consumption among acute medically ill patients in Moscow and Oslo, and the performance of PEth in identifying harmful alcohol use.

**Fig. 1.**
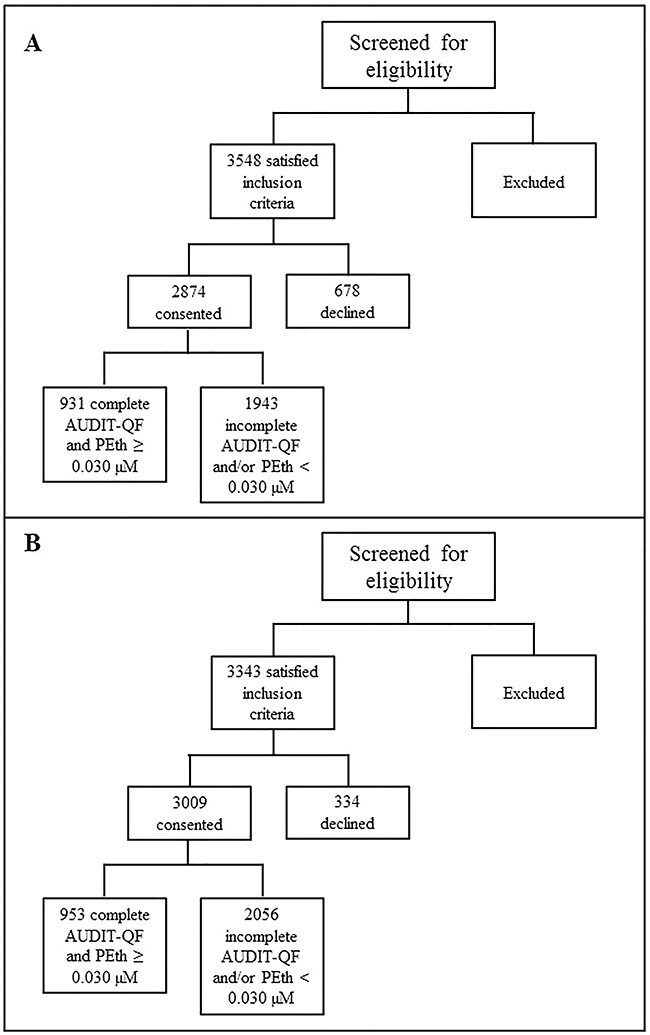
Flow chart of patient recruitment in Oslo (A) and Moscow (B).

## METHODS

### Design and site selection

This comparative study was part of a larger study comprising acute medically ill patients from two urban hospitals in Oslo and Moscow: Lovisenberg Diaconale Hospital (LDS) in Oslo and V.P. Demikhov Hospital 68 in Moscow (Kabashi et al., 2019). The inclusion period lasted from November 2016 to December 2017. In Oslo, patients were recruited by emergency department nurses. The medical wards at the hospital were General Internal Medicine, Infectious Diseases, Cardiology, Pulmonary Medicine, Cerebrovascular and Geriatric conditions, Hematology or Gastroenterology. In Moscow, patients were recruited by physicians serving as dedicated study and recruitment personnel upon arrival at their respective ward, which was one of the following: General Internal Medicine, Cardiology, Pulmonary Medicine or Neurology. Patients admitted for injuries or surgical conditions were not included in the study. Study participation was voluntary, and those patients that were unable to consent upon admission due to reversible or transient causes (such as intoxication or severe illness) were approached at a later time and asked to participate in the study. Exclusion criteria were age < 18 years of age, permanently unable to give informed consent, elective transfer from other hospitals and limited or no ability to read or write the national language. A total of 2874 patients in Oslo and 3009 patients in Moscow were included in the main study, whereof 931 patients in Oslo and 953 patients in Moscow had complete AUDIT-QF data and PEth levels ≥0.030 μM. Figure 1 depicts a flow chart of the patient recruitment.

### Blood analysis

A blood sample from each patient was collected in 5 ml BD-Vacutainer blood collection glass tubes (BD Vacutainer Systems, Franklin, NJ, USA). In Moscow, the samples were collected at the ward within 24 h of arrival, and were stored at 2–6°C during transportation to the laboratory and analyzed the same day as it was collected. In Oslo, the samples were collected upon arrival and then stored at 4°C before analysis, which was performed within 7 days. The samples were analyzed for PEth 16:0/18:1 using the validated UHPLC-MS/MS method described by Berg et al. (2019). This included sample extraction using Solid Liquid Extraction (SLE) plates Isolute 96-well SLE+ plate with 400 μl bed volume from Biotage (Uppsala, Sweden), using 100 μl blood samples from patients, and calibrators and control samples were prepared by using working solutions added to PEth-free whole blood. The extracted samples were analyzed on Agilent instruments in negative electrospray ionization mode. Patients with PEth concentrations ≥0.030 μM were included in this study, as it is difficult to discriminate between low use and abstinence at lower concentrations (Fig. 1). PEth levels ≥0.300 μM were considered excessive alcohol use (Helander et al., 2019).

**Table 1:** Calculation of AUDIT-QF; monthly drinking events (item 1); amount of alcohol in alcoholic units per drinking event (item 2), and conversion to weekly grams of alcohol.

**AUDIT items**	**AUDIT**		**Weekly grams of alcohol calculation**
**Item 1:** ‘*During the last 12 months how often have you consumed alcohol?*’	**AUDIT responses**	**AUDIT score**	**Frequency monthly drinking events**
	Never	0	0
	Monthly or less	1	1
	2–4 times each month	2	3
	2–3 times each week	3	10
	4 times or more each week	4	16
**Item 2:** ‘*How many units of alcohol (a drink, a glass of wine or a bottle of 0,33 L of beer) do you drink on a “typical” day?*’	**AUDIT responses**	**AUDIT score**	**Amount of alcohol** ^*^
	1–2 units	0	19.2 g
	3–4 units	1	44.8 g
	5–6 units	2	70.4 g
	7–9 units	3	102.4 g
	10 units or more	4	128 g
**Calculation weekly grams of alcohol from item 1 and 2:** *(Frequency monthly drinking events × amount alcohol per drinking event) / 4*			

^*^Grams of alcohol calculated using 12.8 g/unit multiplied with the average number of units per drinking episode. For ‘10 or more units’ 10 units were used.

### Questionnaire

Upon enrollment in the study, the patients filled out a questionnaire containing data on gender and alcohol use. Self-reported alcohol use during the last 12 months was measured using the AUDIT-4, a simple and effective questionnaire comprising four items for identifying alcohol use disorders (Gual et al., 2002), and which has been internationally validated (Saunders et al., 1993; Cook et al., 2011). AUDIT-QF was then derived from the AUDIT-4, and AUDIT-QF points ≥ 5 (men) / 4 (women) were defined as harmful alcohol use. The AUDIT-QF scores were used to calculate weekly grams of alcohol consumed (explained below).

### Calculation of weekly grams of alcohol from AUDIT-QF

In order to calculate weekly grams of alcohol consumed, we converted the AUDIT-QF scores using the following assumptions:

AUDIT item 1 (‘During the last 12 months how often have you consumed alcohol?’) was converted into mean number of drinking events per month. AUDIT item 2 (‘During the last 12 months, how many units of alcohol do you drink on a “typical” day when you are drinking?’) was converted into grams of alcohol per drinking event where one unit was defined as 12.8 g (standard drink of 0.33 L beer with 4.5% alcohol by volume (ABV), 0.15 L wine with 12% ABV, 0.075 L liquor with 20% ABV or 0.04 L liquor with 40% ABV). The grams of alcohol were calculated by multiplying the frequency of monthly drinking events (item 1) with grams of alcohol per drinking event (item 2) and dividing it by 4 to get weekly grams of alcohol (Table 1). Weekly grams of alcohol intake ≥350 g were defined as harmful use (Wood et al., 2018).

### Statistical analysis

The data were analyzed using IBM SPSS 25.0 (Armonk, NY), VassarStats (http://vassarstats.net/) and MedCalc5 software (version 19.1.7). Because PEth concentrations were not normally distributed, we applied nonparametric statistics in the analyses. Only patients with PEth levels ≥0.030 μM were included in this study, as we wanted to investigate patients with positive PEth samples. As previously mentioned, there is an increased risk of false positives when including patients with PEth levels <0.030 μM, because it is difficult to discriminate between low consumption and abstinence. In order to investigate the association between PEth concentration and weekly grams of alcohol consumed, we obtained the 75%, 50% and 25% percentiles of PEth concentrations segregated into weekly grams of alcohol (six zones), and also Spearman’s rho for nonparametric correlations, with an α-value of 0.05. The same was done to investigate the association between PEth concentrations and AUDIT-QF scores (0–8 points). For comparison of the correlation between the two study sites, a comparison of correlation coefficients between the Norwegian and Russian samples was done with Fisher z-transformation (Fisher, 1915), using VassarStats. The performance of PEth in identifying harmful alcohol use was evaluated using the area under the receiver operating characteristics (ROC) curve, with weekly grams of alcohol and AUDIT-QF as reference. Cutoffs defining harmful alcohol use were ≥ 5 (men) / ≥ 4 (women) points when AUDIT-QF was used as reference (Kaarne et al., 2010), and ≥350 grams of alcohol when weekly grams of alcohol were used as a reference, as this amount has been shown to reduce life expectancy (Wood et al., 2018). In addition, to evaluate the effectiveness of PEth, the highest Youden’s indices (*J*) were used to derive the most optimal PEth cutpoints at each study site. To evaluate the test accuracy, a comparison of area under the ROC curves for the Norwegian and Russian samples was also performed, using MedCalc5, based on calculations described by Hanley and McNeil (1982, 1983).

## RESULTS

### Participants

There were totally 2874 Norwegian patients included in this study, with a total of 931 Norwegian patients with complete AUDIT-QF data and PEth concentrations ≥0.030 μM. From the Russian data 3009 patients were included in the study, and 953 of these patients had PEth ≥ 0.030 μM and complete AUDIT-QF data.

As shown in Table 2, most Norwegian patients scored between 2 and 5 points on AUDIT-QF, while most Russian patients scored between 1 and 3 points. However, there were somewhat higher proportions of Russian patients among the higher AUDIT-QF scores (6–8 points) compared to Norwegian patients. Segregated by gender, a larger proportion of Norwegian men scored higher (6–8 points) compared to Norwegian women, while most Russian women scored between 0 and 2 points. Among Russian men, the proportions were somewhat equally divided between all AUDIT-QF scores. The proportion of patients above the cutoff for harmful alcohol use (≥ 5 (men) / 4 (women)) were 46.4% in Oslo and 52.1% in Moscow.

**Table 2:** Prevalence of AUDIT-QF scores, weekly grams of alcohol zones and PEth concentration zones among patients in Oslo and Moscow, segregated by gender and total

**AUDIT-QF score**	**Oslo (N = 931)**			**Moscow (N = 953)**		
	**Men** **(N (%))**	**Women** **(N (%))**	**Total** **(N (%))**	**Men** **(N (%))**	**Women** **(N (%))**	**Total** **(N (%))**
**0**	1 (0.2)	0 (0)	**1 (0.1)**	32 (4.8)	30 (10.7)	**62 (6.5)**
**1**	22 (3.8)	17 (4.8)	**39 (4.2)**	40 (6.0)	80 (28.5)	**120 (12.6)**
**2**	67 (11.6)	53 (14.9)	**120 (12.9)**	64 (9.5)	59 (21.0)	**123 (12.9)**
**3**	129 (22.4)	116 (32.7)	**245 (26.3)**	88 (13.1)	38 (13.5)	**126 (13.2)**
**4**	162 (28.1)	94 (26.5)	**256 (27.5)**	97 (14.4)	25 (8.9)	**122 (12.8)**
**5**	95 (16.5)	57 (16.1)	**152 (16.3)**	128 (19.0)	20 (7.1)	**148 (15.5)**
**6**	46 (8.0)	9 (2.5)	**55 (5.9)**	113 (16.8)	15 (5.3)	**128 (13.4)**
**7**	30 (5.2)	8 (2.3)	**38 (4.1)**	52 (7.7)	3 (1.1)	**55 (5.8)**
**8**	24 (4.2)	1 (0.3)	**25 (2.7)**	58 (8.6)	11 (3.9)	**69 (7.2)**
**≥Cutoff 5 (men) / 4 (women)**	357 (62.0)	75 (21.1)	**432 (46.4)**	448 (66.7)	49 (17.4)	**497 (52.1)**
**Weekly grams of alcohol**	**Oslo (N = 931)**			**Moscow (N = 953)**		
**0.0**–**12.8 g**	49 (8.5)	29 (8.2)	**78 (8.4)**	114 (17.0)	154 (54.8)	**268 (28.1)**
**12.8**–**99.9 g**	299 (51.9)	229 (64.5)	**528 (56.7)**	367 (54.6)	97 (34.5)	**464 (48.7)**
**100.0**–**199.9 g**	138 (24.0)	83 (23.4)	**221 (23.7)**	38 (5.7)	5 (1.8)	**43 (4.5)**
**200.0**–**299.9 g**	36 (6.3)	5 (1.4)	**41 (4.4)**	43 (6.4)	11 (3.9)	**54 (5.7)**
**300.0**–**399.9 g**	12 (2.1)	4 (1.1)	**16 (1.7)**	27 (4.0)	0 (0.0)	**27 (2.8)**
**≥400.0 g**	42 (7.3)	5 (1.4)	**47 (5.0)**	83 (12.4)	14 (5.0)	**97 (10.2)**
**≥Cutoff 350 g**	42 (7.3)	5 (1.4)	**47 (5.0)**	102 (15.2)	15 (5.3)	**117 (12.3)**
**PEth concentration zones**	**Oslo (N = 931)**			**Moscow (N = 953)**		
**0.030**–**0.299 μM**	386 (67.0)	268 (75.5)	**654 (70.2)**	344 (51.1)	181 (64.4)	**525 (55.0)**
**≥0.300 μM**	190 (33.0)	87 (24.5)	**277 (29.8)**	329 (48.9)	100 (35.6)	**429 (45.0)**

Divided into weekly grams of alcohol zones, most Norwegian patients reported consuming between 12.8 and 199.9 g alcohol weekly, while the vast majority of Russian patients reported lower alcohol consumption, between 0.0 and 99.9 g alcohol weekly. There were, however, more Russian patients among the higher consumption groups (≥200.0 g) compared to Norwegian patients, and 12.3% of Russian patients scored above the cutoff for harmful alcohol use (≥350 g), compared to 5.0% of Norwegian patients.

Divided by PEth concentrations, there were higher proportions of excessive drinkers (≥0.300 μM) among men (Oslo: n = 190, 33.0%. Moscow: n = 329, 48.9%) compared to women (Oslo: n = 87, 24.5%. Moscow: n = 100, 35.6%) in each country.

### Relationship between PEth concentrations and self-reported alcohol consumption

Association between PEth and weekly grams of alcohol was investigated using 75%, 50% and 25% percentiles of PEth concentrations within AUDIT-QF scores and within weekly grams of alcohol zones.

**Fig. 2.**
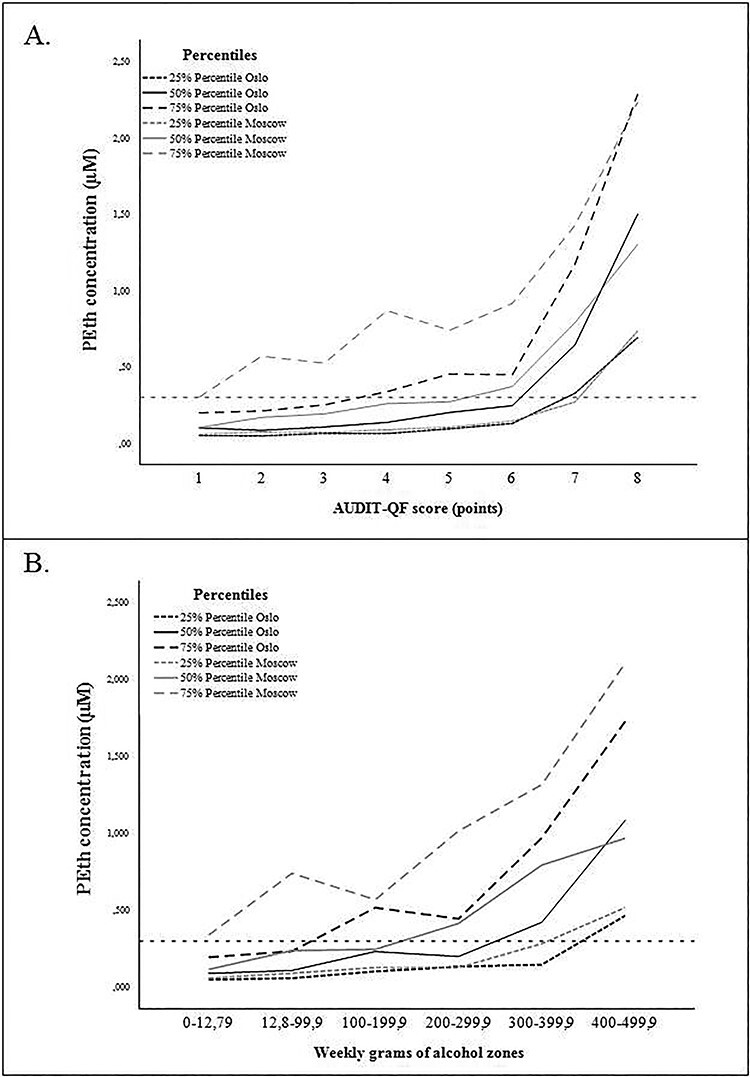
75th, 50th and 25th percentiles of PEth concentrations within AUDIT-QF scores (A) and weekly grams of alcohol zones (B).

Figure 2A depicts the association between PEth concentrations and AUDIT-QF total score as 75%, 50% and 25% percentiles. It shows that for patients with AUDIT-QF scores ≤6, all of the Norwegian patients within the 75% percentile had PEth levels below 0.500 μM, and exponentially increasing PEth levels with increasing AUDIT-QF scores. Among the Russian patients, we found more variation in PEth concentrations within the 75% percentile across the AUDIT-QF scores, but with a generally increasing trend. The increases in PEth concentrations were steeper from 6 to 8 points in both countries. The median PEth concentrations (50% percentile) in the data from Oslo and Moscow were somewhat similar across patients with the same AUDIT-QF scores, although with slightly higher concentrations among the Russian patients with lower scores compared to the Norwegian patients with similar scores. However, among patients with AUDIT-QF score of 8 the Norwegian patients had higher median PEth concentrations compared to the Russian patients.

Figure 2B depicts the association between PEth concentrations and weekly grams of alcohol calculated from AUDIT-QF. The median PEth concentrations were generally higher among the Russian patients, and PEth reached 0.300 μM at self-reported lower alcohol use, compared to the Norwegian patients. Compared to the association between PEth and AUDIT-QF total score, there was a larger median difference between the two patient groups in the association between PEth and weekly grams of alcohol.

Ideally, it would be informative to investigate gender-differences in the association between PEth and self-reported alcohol intake, however, because of the relatively low number of women from either site reporting high alcohol intake, this was not possible.

A Spearman’s correlation was run to determine the relationship between PEth concentrations and AUDIT-QF scores in patients with PEth concentrations above 0.030 μM. A weak, positive correlation was found in the Norwegian sample (*r_s_* = 0.326, n = 931, *P* < 0.001), and the Russian sample (*r_s_* = 0.366, n = 953, *P* < 0.001). From Fisher *z*-transformation, we found no significant difference between the correlation coefficients in Oslo and Moscow (*z* = −0.98, two-tailed *P* = 0.327).

A Spearman’s correlation was also calculated between PEth concentrations and weekly grams of alcohol in patients with PEth concentrations above 0.030 μM, and a weak, positive correlation was found in the Norwegian sample (*r_s_* = 0.354, n = 931, *P* < 0.001), and the Russian sample (*r_s_* = 0.339, n = 953, *P* < 0.001). There was no significant difference between the correlation coefficients in Oslo and Moscow (z = 0.47, two-tailed *P* = 0.638).

**Fig. 3.**
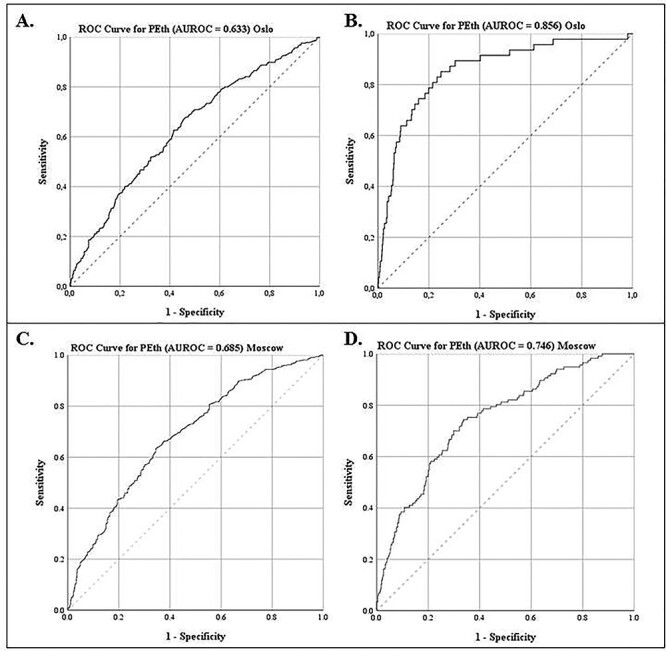
ROC curves for PEth predicting harmful alcohol use by AUDIT-QF (cutoff ≥ 5 (men) / ≥ 4 (women)) (A and C) and weekly grams of alcohol (cutoff ≥ 350 g) (B and D) (top panel: Oslo, bottom panel: Moscow). A. Predictive accuracy of PEth (≥0.030 μM) with area under the ROC curve of 0.633 for harmful alcohol consumption using AUDIT-QF as reference (Oslo). B. Predictive accuracy of PEth (≥0.030 μM) with an area under the ROC curve of 0.856 for harmful alcohol consumption using weekly grams of alcohol as reference (Oslo). C. Predictive accuracy of PEth (≥0.030 μM) with area under the ROC curve of 0.685 for harmful alcohol consumption using AUDIT-QF as reference (Moscow). D. Predictive accuracy of PEth (≥0.030 μM) with an area under the ROC curve of 0.746 for harmful alcohol consumption using weekly grams of alcohol as reference (Moscow).

The area under the ROC curve for PEth as a continuous variable was 0.633 (95% CI: 0.596, 0.669) when using AUDIT-QF ≥ 5 (men) / 4 (women) as cutoff for harmful alcohol use for the Norwegian patients (Fig. 3A), and the optimal PEth cutpoint for discrimination between non-harmful and harmful alcohol use was 0.128 μM (Youden’s *J* = 0.217). For the Russian patients the area under the ROC curve for PEth was 0.685 (95% CI: 0.651, 0.718, *P* < 0.001, Fig. 3C), and the optimal cutpoint was 0.270 μM (Youden’s *J* = 0.289). We found a significant difference in the area under the curve between the Norwegian and the Russian samples at −0.052 (two-tailed *P* = 0.043). The area under the ROC curve for PEth as a continuous variable was 0.856 (95% CI: 0.798, 0.914) when using weekly grams of alcohol ≥ 350 grams for the Norwegian patients (Fig. 3B), and the optimal PEth cutpoint for discrimination between non-harmful and harmful alcohol was 0.327 μM (Youden’s *J* = 0.603). For the Russian patients the area under the curve was 0.746 (95% CI: 0.700, 0.793, *P* < 0.001 Fig. 3D), with the most optimal cutpoint being 0.396 μM (Youden’s *J* = 0.408). There was a significant difference between the area under the curve in the Norwegian and Russian samples (0.11, two-tailed *P* = 0.013).

## DISCUSSION

Our study showed that self-reported alcohol intake using AUDIT-QF correlated positively with PEth levels in blood, but converting AUDIT-QF into weekly grams of alcohol consumed resulted in better discrimination of PEth for harmful alcohol use. Thus, our findings propose that PEth can be used to identify patients with harmful alcohol consumption. By converting AUDIT-QF scores to weekly grams of alcohol it is possible to get an estimate of the amount of alcohol consumed.

From both AUDIT-QF scores and weekly grams of alcohol zones, it was found that the majority of patients from either country do not have harmful alcohol consumption. However, the distribution of reported alcohol consumption varies between the two countries, with higher proportions of Russian patients reporting both low and high consumption compared to Norwegian patients, which generally reports medium consumption. When divided into gender, we found that Russian women generally reported lower alcohol consumption compared to Russian men, which might be explained by traditional gender roles and culture. One study found large gender differences in drinking patterns in the Russian city of Novosibirsk, explained in large by different expectations toward drinking for men and women (Bobrova et al., 2010). Russian women also reported lower consumption compared to Norwegian women, but had higher proportion of patients with excessive alcohol use (PEth levels ≥ 0.300 μM), which might indicate underreporting among Russian women because of social desirability. Likewise, in a study by Laatikainen et al. (2002), where they investigated self-reported alcohol consumption and alcohol biomarkers among women and men in the Republic of Karelia, Russia and in North Karelia, Finland, they found higher degree of underreporting among Russian women compared to Finnish women (Laatikainen et al., 2002). From the percentiles of PEth concentrations across AUDIT-QF scores and weekly grams of alcohol zones, the Norwegian patients generally had lower median PEth concentrations compared to Russian patients with the same scores and zones, which might reflect a generally lower consumption among the Norwegian patients or a different drinking pattern between the two sites.

PEth gave a better discrimination of harmful consumption when using weekly grams of alcohol ≥350 g as reference, as shown by the area under the ROC curves, compared to using AUDIT-QF as reference. There was a significant difference when comparing area under the ROC curves between the Norwegian and Russian patients, using both AUDIT-QF and weekly grams of alcohol as reference, indicating differences in test accuracy between the two countries, with better performance of AUDIT-QF in Moscow, and weekly grams of alcohol in Oslo. When using AUDIT-QF as reference, PEth cutpoints of 128 μM in Oslo and 270 μM in Moscow provided the most optimal discrimination of harmful alcohol use. This suggest that the AUDIT-QF cutoff (5 (men) / 4 (women)) in Norway might be too low, considering that PEth levels ≥0.300 μM are indicative of excessive drinking. Interestingly, the variation in PEth cutpoints between the countries decrease and are closer to expected PEth levels for excessive alcohol use when AUDIT-QF is transformed into weekly grams of alcohol. This difference might be due to a more accurate calculation of alcohol consumption when using weekly grams of alcohol, and probably yields a higher cutoff compared to AUDIT-QF.

When compared to previous studies of AUDIT and PEth in hospital settings, our findings are generally in line with these. In a study with patients admitted to a Burns Unit and medical intensive care hospital wards, inpatient alcohol detoxification patients and controls, PEth showed excellent area under the ROC curve at 0.948 when using AUDIT-C as reference (Afshar et al., 2017). In a similar study, Kip et al. (2008) demonstrated that PEth produced an area under the ROC curve of 0.672 when using AUDIT as reference in medical emergency patients. However, patients with illicit drug use, elevated blood alcohol concentration and liver disease were excluded from that study, which might have biased the results. In a study of alcohol-dependent patients, where the patients were screened with AUDIT-C and retrospective self-reported alcohol consumption (last 60 days) upon admission and daily alcohol consumption and PEth during the 6 week study period, PEth was found to be weakly correlated with retrospective self-reported consumption at baseline (r*_s_* = 0.23, *P* < 0.05), but not with AUDIT-C. Better correlations were found between PEth and self-reported daily alcohol consumption (Walther et al., 2015), indicating that reported daily alcohol consumption is more reliable compared to retrospective self-report. The findings from these studies and our study indicate that AUDIT is sensitive in identifying harmful alcohol use, but data on consumption may be an even better measure. The difference in predictive value of AUDIT-QF and weekly grams of alcohol between the two countries in our study might reflect a difference in drinking pattern or bias in self-reporting, and we can only assume this will vary between other countries as well. In addition, our data show that Norwegian patients scored higher on frequency (item 1), while Russian patients scored higher on quantity (item 2), reflecting different drinking pattern, but resulting in the same AUDIT-QF score. We know that long bouts of continuous heavy drinking alternating with days of abstinence, known as zapoi, is more common in Russia (Tomkins et al., 2007; Shield and Rehm, 2015), while single-occasion heavy drinking is more common in Norway (SIRUS, 2016).

Although PEth appears to be a stable biomarker for long-term alcohol use, there are some indications that formation and elimination rate might be affected by drinking pattern, which can explain some of the discrepancy between self-reported alcohol use and PEth concentrations in our results. Previous studies have shown that for social drinkers (≤2–3 alcohol units/day), the PEth elimination rate appears to be somewhat slower compared to heavy consumers, with half-life of 4.5–10.1 days the first week after sobriety, and 5-12 days the second week of sobriety (Gnann et al., 2012). There might be several explanations for the different PEth-formation and elimination rates, such as individual differences in alcohol absorption and phospholipase D activity (Hahn et al., 2016), and also formation of PEth *in vitro* in the presence of ethanol (Schrock et al., 2018). Several studies have found some correlation between amount of alcohol consumed by self-report and PEth concentrations (Varga et al., 2000; Wurst et al., 2004, 2010; Aradottir et al., 2006). In a study where alcohol was administered to healthy individuals (47 g for men, 32 g for women), PEth was not detected within the first day after intake (Varga et al., 1998). However, as Ulwelling and Smith (2018) discuss in a review article from 2018, there are several factors that can affect the interpretation of the association between PEth and alcohol consumed, namely the length of abstinence before consumption, whether consumption was self-reported or administered, and whether consumption was measured in quantity or dosed according to body weight, to mention some (Ulwelling and Smith, 2018). It may be theorized that the patients in our study, with otherwise high alcohol consumption, might have stopped drinking several days before admittance to hospital because of their acute medical condition, resulting in lower PEth concentrations.

AUDIT-QF was calculated to assess alcohol consumption in our study. AUDIT-C is, however, more commonly used in various settings, including health care (Reinert and Allen, 2007). The first question focuses on frequency of drinking, the second question about quantity, and the third question about binge drinking. AUDIT-QF does not include the question on binge drinking which is crucial when the aim is to assess if the patients alcohol use is harmful. AUDIT-QF considers the quantity and frequency of alcohol intake, which was considered more accurate when the aim was to compare the intake of alcohol to the formation of PEth. AUDIT-C is not accurate in discriminating quantity because of the latitude in response categories, and because alcohol-related health risks are dose-dependent, it would be beneficial to know more exactly how many drinks each individual consumes each week. An alternative to AUDIT-C could be to use other questionnaires on alcohol consumption, one being daily drinking estimation (DDE) questionnaires, such as Timeline Followback (TLFB) (Sobell and Sobell, 2003), where the responders are asked to retrospectively recall their daily alcohol consumption ranging from 1 to 12 months prior. However, a major limitation is that this is more time-consuming, which is not always feasible in a clinical setting.

The amount of alcohol in one unit differs in different countries. In Norway a standard drink is 12.8 g, while in Russia it is 10 g. To make comparisons, the size of the standard drink was set to 12.8 g at both sites. The rationale was that this corresponds better with the actual size of alcoholic beverages sold; it is however considered a limitation which might inflate the self-reported intake of grams of alcohol among the Russian patients.

Because of the latitude in the response categories of AUDIT-QF, especially in the last response alternative of item 2 (‘10 or more units’), we chose to use 10 units for all patients that answered this. However, we believe the mean value will be higher, since some heavy consumers drink more than 10 units on single occasions, although it would be impossible to speculate on the real mean value in our sample.

Although PEth is a relatively stable biomarker for ethanol consumption, it is affected by sample storage temperature. In our study, the blood samples were sampled and analyzed the same day in Moscow and within 7 days in Oslo, which might have resulted in different degradation of PEth at the two sites. In addition, it was not possible to send and cross-validate the blood samples between the sites, which could have helped detect possible analytical variations between the laboratories. Comparison of PEth with other established ethanol biomarkers would be beneficial to test the performance of PEth in regards to alcohol use. However, this was not feasible in this study, which limits the generalizability of our findings.

The comparison between self-reported alcohol consumption and PEth has shown a relatively good correlation, and the use of PEth to complement other assessment of alcohol consumption both in research and clinical practice seems promising. More studies in other populations are warranted to assess the generalizability of these correlations. A possible approach to enhance the accuracy of identifying patients with harmful alcohol use in clinical settings could be to screen all patients using AUDIT-QF, given the good performance in identifying patients with a potentially harmful alcohol use, and continue with analyzing PEth combined with a short-form questionnaire on self-reported alcohol consumption the previous 30 days for a more accurate determination of harm level.

The AUDIT questionnaire is used as a screening tool to identify harmful alcohol use, without quantifying alcohol use. Likewise, PEth is currently used as a marker for alcohol use, with PEth levels ≥20 ng/ml (0.028 μM) indicating alcohol intake (Walther et al., 2015), and PEth levels ≥0.300 μM indicating harmful pattern (Helander and Hansson, 2013). However, as demonstrated by Wood et al. (2018), even low amounts of alcohol use have health consequences. As shown in this study, PEth corresponds well with self-reported drinking, and may subsequently be used as an estimate of alcohol consumption, which is of importance in clinical settings. But because there is a discrepancy in time interval between AUDIT (reports on alcohol use the previous 12 months) and PEth (reflects long term alcohol consumption, especially within the last 2 weeks before sampling (Helander et al., 2019), the correlation might be biased.

Future studies should assess PEth concentrations in correlation to more accurate self-reported alcohol consumption questionnaires in a time-frame closer to PEth measurements, as this would provide a better correlation and understanding of the relationship between consumed alcohol and PEth.

## Data availability

Data cannot be shared, due to an institutional agreement.

## Funding

The work was supported by grant B-1408 on Norwegian-Russian collaboration in health and social issues provided by the Ministry of Health and Care Services, Oslo, Norway. The Ministry of Health and Care Services had no role in the study design, in the collection, analyses or interpretation of the data, in the writing of the report or the decision to submit the article for publication.

## Conflict of interest statement

No conflict of interest declared.
